# L- and D-lactate enhance DNA repair and modulate the resistance of cervical carcinoma cells to anticancer drugs via histone deacetylase inhibition and hydroxycarboxylic acid receptor 1 activation

**DOI:** 10.1186/s12964-015-0114-x

**Published:** 2015-07-25

**Authors:** Waldemar Wagner, Wojciech M. Ciszewski, Katarzyna D. Kania

**Affiliations:** Laboratory of Cellular Immunology, Institute of Medical Biology, Polish Academy of Science, Lodz, Poland; Laboratory of Transcriptional Regulation, Institute of Medical Biology, Polish Academy of Science, Lodz, Poland

**Keywords:** Lactate, DNA repair, HDACs, HCAR1, Cervical cancer

## Abstract

**Background:**

The consideration of lactate as an active metabolite is a newly emerging and attractive concept. Recently, lactate has been reported to regulate gene transcription via the inhibition of histone deacetylases (HDACs) and survival of cancer cells via hydroxycarboxylic acid receptor 1 (HCAR1). This study examined the role of L- and D-lactate in the DNA damage response in cervical cancer cells.

**Methods:**

Three cervical cancer cell lines were examined: HeLa, Ca Ski and C33A. The inhibitory activity of lactate on HDACs was analysed using Western blot and biochemical methods. The lactate-mediated stimulation of DNA repair and cellular resistance to neocarzinostatin, doxorubicin and cisplatin were studied using γ-H2AX, comet and clonogenic assays. HCAR1 and DNA repair gene expression was quantified by real-time PCR. DNA-PKcs activity and HCAR1 protein expression were evaluated via immunocytochemistry and Western blot, respectively. HCAR1 activation was investigated by measuring intracellular cAMP accumulation and Erk phosphorylation. *HCAR1* expression was silenced using shRNA.

**Results:**

L- and D-lactate inhibited HDACs, induced histone H3 and H4 hyperacetylation, and decreased chromatin compactness in HeLa cells. Treating cells with lactate increased *LIG4*, *NBS1*, and *APTX* expression by nearly 2-fold and enhanced DNA-PKcs activity. Based on γ-H2AX and comet assays, incubation of cells in lactate-containing medium increased the DNA repair rate. Furthermore, clonogenic assays demonstrated that lactate mediates cellular resistance to clinically used chemotherapeutics. Western blot and immunocytochemistry showed that all studied cell lines express HCAR1 on the cellular surface. Inhibiting HCAR1 function via pertussis toxin pretreatment partially abolished the effects of lactate on DNA repair. Down-regulating HCAR1 decreased the efficiency of DNA repair, abolished the cellular response to L-lactate and decreased the effect of D-lactate. Moreover, HCAR1 shRNA-expressing cells produced significantly lower mRNA levels of monocarboxylate transporter 4. Finally, the enhancement of DNA repair and cell survival by lactate was suppressed by pharmacologically inhibiting monocarboxylate transporters using the inhibitor α-cyano-4-hydroxycinnamic acid (α-CHCA).

**Conclusions:**

Our data indicate that L- and D-lactate present in the uterine cervix may participate in the modulation of cellular DNA damage repair processes and in the resistance of cervical carcinoma cells to anticancer therapy.

**Electronic supplementary material:**

The online version of this article (doi:10.1186/s12964-015-0114-x) contains supplementary material, which is available to authorized users.

## Introduction

The model of lactate as an active metabolite has emerged as an attractive concept. The role of lactate as a signalling factor is supported by observations that lactate mimics hypoxic conditions, stimulates connective tissue synthesis and enhances endothelial cell mobility and tumour angiogenesis [1–3]. Locally produced L-lactate may play a role in hormone function in an autocrine and paracrine fashion via hydroxycarboxylic acid receptor 1 (HCAR1, also referred to as GPR81/FKSG80) to exert antilipolytic effects on adipose tissue [4] or to modulate the activity of primary cortical neurons [5]. Recently, HCAR1 has been implicated in the regulation of lactate transport mechanisms. HCAR1 presence enhances pancreatic cancer cell growth and metastasis [6], and is necessary for survival of the HER2-positive and the triple-negative breast cancer cells [7]. The intracellular biological activity of lactate depends on its cellular uptake, which is facilitated by monocarboxylate transporters (MCTs) [2]. Functional studies have identified MCT1-4 in the plasma membrane of various cell types, including uterine cervical cells, and have demonstrated that the bidirectional transport of monocarboxylates (*e.g.*, lactic acid, pyruvic acid and acetic acid) across the plasma membrane is directed by substrate and proton concentration gradients [8]. Cells with high glucose metabolism (*e.g.*, most cancer cells) export lactic acid to maintain intracellular homeostasis, whereas other cells, such as astrocytes and heart and skeletal muscle cells, import lactic acid for mitochondrial respiration or as a substrate for gluconeogenesis (hepatocytes). The weak inhibitory activity of both L- and D-lactate on histone deacetylases (HDACs) was also recently reported [9]. Thus, lactate, a natural fermentation product (e.g., butyrate, an established potent HDAC inhibitor), is an important effector of the epigenetic regulation of chromatin function. HDACs are involved in acetylation, an important posttranslational protein modification, and their activity opposes that of histone acetyltransferases (HATs). In general, an increasing level of histone acetylation (hyperacetylation) results in a more relaxed, transcriptionally permissive chromatin conformation, whereas the reverse action (hypoacetylation) results in a more condensed, transcriptionally repressive chromatin state. One of the primary implications of chromatin-HAT/HDAC remodelling complex interactions is its important but poorly characterised role in regulating the DNA damage response (DDR) [10, 11]. HATs and HDACs are recruited to DNA double-strand break (DSB) sites to create a repair-proficient chromatin state that orchestrates the activity of repair and signalling proteins, thereby promoting DNA repair processes [12–14]. Furthermore, recent evidence suggests that the coordinated action of HATs/HDACs may directly affect the DDR by modulating the activity of key proteins involved in DNA damage detection and repair, such as DNA-PK [15] and ATM [16].

The lower female genital tract is an internal structure of the body in which an extremely high concentration of lactate is maintained by symbiotic lactic acid bacteria under physiological conditions [17]. Vaginal secretions may contain 10–50 mM lactate; approximately 55 % of vaginally secreted lactate is the D isoform [18]. Therefore, the modulation of mucosal epithelial cell activity in the female reproductive tract via the inhibition of HDACs by lactate represents an appealing topic of investigation because these cells are constantly exposed to L-/D-lactate of bacterial origin. This potential modulation is particularly important in the context of patients with cervical cancer, in which lactate may modulate the activity of the cervical cancer cells in a manner that alters the effectiveness of chemotherapeutic agents. The present study reports a novel biological activity of lactate, specifically the modulation of cellular DDR processes, in cervical cancer cells. We demonstrated that concentrations of L- and D-lactate consistent with those observed in the uterine cervix inhibit class I and II HDACs, induce the hyperacetylation of H3 and H4 histones, increase chromatin accessibility and significantly enhance the DNA repair rate in cervical cancer cells, as evaluated by γ-H2AX and comet assays. The observed increase in the activity of the DNA repair machinery was accompanied by a significant enhancement of the survival of three different cervical cancer cell lines after chemotherapeutic treatment. In addition, we showed that all three examined cervical cancer cell lines display surface expression of HCAR1, which is known to be involved in cell survival. Furthermore, we demonstrated the essential role of HCAR1 and MCTs in the lactate-mediated enhancement of cellular DNA repair capacity and in the resistance of the examined cervical cancer cell lines to anticancer chemotherapeutics. Importantly, the present study provides new insight into the role of microorganism-mammalian cell interactions in the female genital tract and demonstrates a novel mechanism underlying the regulation of cellular resistance to genotoxins/chemotherapeutics.

## Results

### L- and D-lactate stimulate the acetylation of histones *via* the inhibition of histone deacetylases

**Fig. 1:**
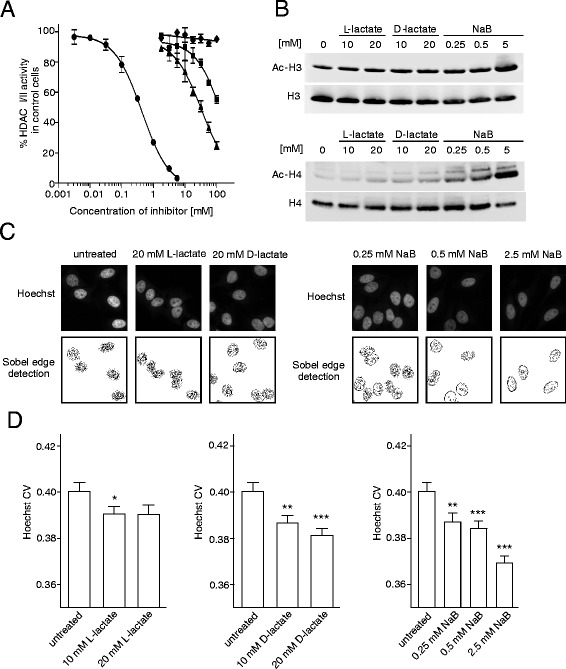
Lactate induces histone hyperacetylation and alters chromatin compactness by inhibiting HDACs. **a** HeLa cells were treated with increasing concentrations of L-lactate, D-lactate, butyrate or NaCl for 2 h before measuring cellular HDAC activity. Basal HDAC activity was subtracted, and the value observed in the control cells was treated as 100 %. The graph displays the mean ± SEM of HDAC activity from three independent experiments in the presence of butyrate (*black circles*), L-lactate (*black squares*), D-lactate (*black triangles*) or NaCl (control, *black diamonds*). **b** Histone H3 and H4 acetylation in HeLa cells after 24 h of treatment with L-lactate, D-lactate or butyrate was detected via Western blot analysis. Representative blots of three independent experiments are shown. **c**, **d** The effect of lactate and butyrate on chromatin compactness. HeLa cells were treated with the indicated concentration of L-lactate, D-lactate or butyrate for 24 h before fixation and Hoechst 33342 staining. Then, the cells were analysed using an ArrayScan VTI HCS Reader. **c** Hoechst staining of DNA in representative nuclei and corresponding images after Sobel edge detection transformation. **d** Graphs of the CVs for Hoechst fluorescence intensities for all pixels within each nucleus. The CVs are presented as the means ± SEM from four independent experiments. Statistical significance was evaluated using one-way ANOVA followed by Tukey’s test. *P < 0.05, **P < 0.01 and ***P < 0.001 indicate significant differences compared to the untreated cells

Both L- and D-lactate inhibit HDACs in cell-free extracts [9]. Here, we examined the effect of lactate on HDAC activity in live cells. Sodium butyrate, an established HDAC inhibitor, was used as a positive control. D-lactate more potently inhibited cellular HDAC activity than L-lactate (Fig. 1a). The IC_50_ values for L-lactate, D-lactate, and butyrate were 124 ± 12, 32 ± 4, and 0.40 ± 0.01 mM, respectively, and were 4-fold (lactate) to 8-fold (butyrate) higher than the IC_50_ values obtained for nuclear protein extracts *in vitro* [9]. Next, we determined whether lactate induces histone hyperacetylation in cultured HeLa cells *in vivo*. Both histones H3 and H4 were acetylated after treatment with lactate or butyrate in a concentration-dependent manner (Fig. 1b). The effects of 20 mM L- or D-lactate, the physiological concentration of lactate in the uterine cervix, on HDAC inhibition and histone hyperacetylation indicated its relatively weak activity, as its butyrate equivalent activity was calculated as a concentration of 0.25–0.5 mM.

### Lactate- and butyrate-induced histone hyperacetylation corresponds to decreased chromatin compactness

Increased histone acetylation at lysine residues due to the dominance of HAT activity over HDAC activity reduces the positive charge of histones and disrupts electrostatic interactions between DNA and histones. Extensive acetylations occur on numerous histone tail lysines, including H3K9, H3K14, H3K18, H4K5, H4K8 and H4K12 [19]. This process leads to chromatin relaxation. Because both lactate and butyrate are HDAC inhibitors that contribute to H3 and H4 pan-acetylation, we examined the effect of lactate on chromatin compactness. We studied lactate and butyrate at concentration ranges of 10–20 mM and 0.25–2.5 mM, respectively. After 24 h of treatment, we observed a decrease in chromatin compactness for both compounds tested, which was observed as a reduction in the number of distinct spaces within the nucleus (Fig. 1c) after image transformation using the Sobel edge detection algorithm. Raw images of Hoechst-stained cells were used to calculate the coefficient of variation (CV) for Hoechst nuclear fluorescence and to quantify chromatin compactness (Fig. 1d). Incubating cells with 2.5 mM butyrate significantly decreased the Hoechst CV by 8 %, whereas cells treated with 20 mM L-lactate or D-lactate showed a less pronounced decrease in the Hoechst CV, as their respective Hoechst CVs were 3.2- and 1.7-fold lower than that of 2.5 mM butyrate.

### L- and D-lactate enhance DNA double-strand break repair following neocarzinostatin, doxorubicin and cisplatin treatment

**Fig. 2:**
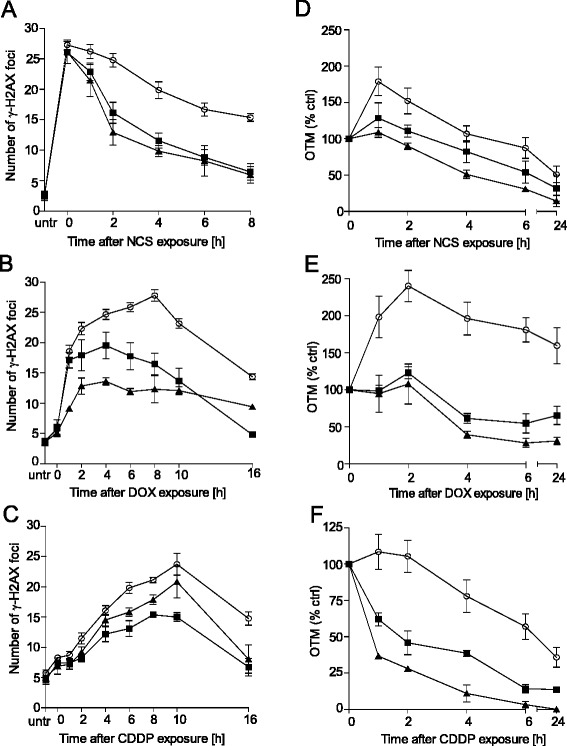
L- and D-lactate treatment increases DSB repair in cells treated with chemotherapeutics. HeLa cells were incubated in the presence or absence of 20 mM L- or D-lactate for 24 h, followed by treatment with NCS, DOX or CDDP for 30 min. Then, the cells were allowed to recover for the indicated period. **a**, **b**, **c** γ-H2AX foci resolution kinetics after chemotherapeutic treatment (2 nM NCS, 2 μM DOX or 20 μM CDDP). The graphs show the mean number of γ-H2AX foci per cell ± SEM from at least three independent experiments; drug alone (*white circles*), L-lactate + drug (*black square*), D-lactate + drug (*black triangles*). **d**, **e**, **f** DDR dynamics after chemotherapeutic treatment (5 nM NCS, 2 μM DOX or 20 μM CDDP) as measured by a neutral comet assay. The basal Olive tail moment (OTM) was subtracted, and the value observed at 0 h was set to 100 %. The graph shows the mean OTM (% of control) ± SEM from three independent experiments; drug alone (*white circles*), L-lactate + drug (*black square*), D-lactate + drug (*black triangles*)

Pre-existing histone H3 and H4 modifications can directly influence the cellular DDR. We studied the kinetics of γ-H2AX foci formation, a surrogate marker of DNA DSBs, after exposure to doxorubicin (DOX), cisplatin (CDDP) or neocarzinostatin (NCS, radiomimetic) to determine the role of lactate in the cellular DDR. In response to DNA DSBs, H2AX histones become phosphorylated and form γ-H2AX histones, which can be detected as nuclear foci via immunofluorescence. The kinetics of γ-H2AX foci formation were determined after the treatment of HeLa cells with lactate (either L- or D-lactate) for 24 h. Cells were then exposed to a chemotherapeutics for 30 min, followed by measurement of the resolution kinetics of the foci for up to 16 h. Both L- and D-lactate significantly increased the resolution of γ-H2AX foci after treatment with all three chemotherapeutics (Fig. 2a-c and Additional file 1, Additional file 2, Additional file 3). Interestingly, D-lactate enhanced the disappearance of γ-H2AX foci compared to L-lactate in NCS- and DOX-treated cells, whereas the reverse effect was observed in CDDP-treated cells. We next examined whether the observed enhancement of γ-H2AX foci resolution after lactate treatment was accompanied by increases in the dynamics of DNA DSB repair. We performed comet assays under conditions similar to those of the γ-H2AX assay. Both L- and D-lactate significantly enhanced DNA repair after chemotherapeutic treatment, and the greatest enhancement was observed after treatment with DOX and CDDP (Fig. 2d-f and Additional file 4, Additional file 5, Additional file 6). Furthermore, the effects of D-lactate on DNA repair dynamics were greater than those of L-lactate for all studied DNA-damaging agents.

### Incubating HeLa cells in L- or D-lactate for 24 h induces the expression of genes involved in DNA repair

Because incubating cells with lactate led to a less compact chromatin structure and presumably facilitated DNA access to the transcriptional machinery, we investigated the effects of L- and D-lactate on DNA repair enzyme gene expression, which may have contributed to the observed improvement in the DNA repair rate. We analysed the expression profiles of the following 23 genes associated with primary DNA repair mechanisms: base excision repair (*LIG3*, *XRCC1*, *PNKP*, *PARP1*, and *PARP2*), homologous recombination (HR) (*RAD51*, *BRCA1*, *BRCA2*, *RAD50*, *MRE11A* and *NBS1*), non-homologous end joining (NHEJ) (*XRCC6*, *XRCC5*, *PRKDC*, *LIG4*, *XRCC4*, *DCLRE1C*, *WRN*, *NHEJ1*, *APTX*, and *PARD3*) and DDR (*ATM*, *ATR*, *TP53*, and *MDC1*). Incubating cells with lactate for 24 h exerted notable effects on the mRNA level of three genes: *NBS1*, *LIG4* and *APTX* (Table 1); however, the expression levels of the other genes remained unchanged. L-lactate (D-lactate) significantly increased the expression of *NBS1*, *LIG4* and *APTX* by 1.6 (1.9)-, 1.9 (2.1)- and 1.8 (1.5)-fold, respectively.

**Table 1:** Effect of L-lactate and D-lactate on DNA repair gene expression^a^

Gene	L-lactate	D-lactate
*LIG3*	1.36 ± 0.23	1.13 ± 0.13
*XRCC1*	0.95 ± 0.19	0.81 ± 0.13
*PNKP*	1.22 ± 0.27	1.24 ± 0.13
*PARP1*	1.07 ± 0.29	0.95 ± 0.23
*PARP2*	1.31 ± 0.31	1.00 ± 0.29
*RAD51*	1.06 ± 0.25	0.93 ± 0.18
*BRCA1*	1.12 ± 0.10	1.08 ± 0.19
*BRCA2*	1.16 ± 0.17	1.19 ± 0.30
*RAD50*	1.20 ± 0.20	1.14 ± 0.24
*MRE11A*	0.82 ± 0.09	0.81 ± 0.14
*NBS1*	1.60 ± 0.24*	1.88 ± 0.30**
*XRCC6*	1.01 ± 0.23	0.80 ± 0.26
*XRCC5*	1.12 ± 0.19	1.01 ± 0.30
*PRKDC*	1.02 ± 0.28	1.12 ± 0.41
*LIG4*	1.91 ± 0.17*	2.07 ± 0.46**
*XRCC4*	0.98 ± 0.21	0.93 ± 0.27
*DCLRE1C*	1.13 ± 0.16	0.88 ± 0.19
*WRN*	0.92 ± 0.22	0.73 ± 0.26
*XLF*	1.02 ± 0.16	0.96 ± 0.10
*ATM*	1.13 ± 0.22	0.96 ± 0.30
*ATR*	1.02 ± 0.12	0.88 ± 0.25
*TP53*	0.91 ± 0.16	0.83 ± 0.15
*APTX*	1.76 ± 0.28*	1.54 ± 0.26
*PARD3*	1.01 ± 0.18	1.02 ± 0.18
*MDC1*	1.04 ± 0.24	1. 54 ± 0.82

^a^HeLa cells were treated or not treated with 20 mM L-lactate or 20 mM D-lactate for 24 h before harvesting for RNA isolation. Real-time PCR was performed as described in the Materials and Methods section. The data are presented as the mean fold-changes in gene expression ± SEM of treated cells relative to untreated control cells from at least three independent experiments. All gene expression levels were normalised to the expression of the housekeeping genes *HMBS* and *HPRT* before calculating the expression ratios. Statistical significance was evaluated using one-way ANOVA followed by Tukey’s test. *P < 0.05 and **P < 0.01 denote statistical significance compared to the untreated cells

### Lactate initiates the nuclear activation of DNA-PKcs

**Fig. 3:**
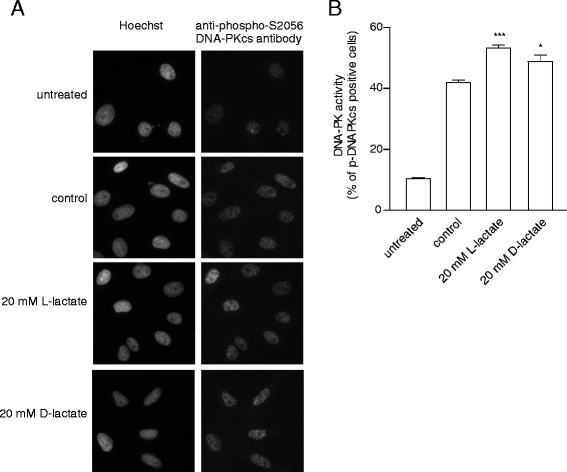
Lactate treatment initiates DNA-PKcs activation. HeLa cells were incubated in the presence or absence of L-lactate or D-lactate for 24 h, exposed to NCS (2 nM) for 30 min, and allowed to recover for 4 h before staining with a phospho-specific antibody directed against Ser2056 of DNA-PKcs. **a** Immunocytochemical staining of DNA-PKcs phosphorylation at S2056. Each image shows representative microscopic area for the particular treatment from the same experiment. **b** The graphs show the means ± SEM of the percentage of cells containing more than six foci from three independent experiments. Statistical significance was evaluated using one-way ANOVA followed by Tukey’s test.*P < 0.05, **P < 0.01 and ***P < 0.001 indicate significant differences compared to the untreated cells

Chromatin affects DNA accessibility and provides docking sites for repair and signalling proteins. Recent evidence has suggested that epigenetic modifications to chromatin, particularly H3 acetylation, initiate the activation of DNA-PKcs and its relocalisation from a soluble nucleoplasmic compartment to a less extractable nuclear fraction [20]. DNA-PKcs is the key enzyme involved in NHEJ, which is a DSB repair pathway that is active throughout the cell cycle (HR pathway is restricted to the S and G2 phases). Thus, we evaluated the level of DNA-PKcs phosphorylation upon NCS treatment. The induction of DNA DSBs promoted the phosphorylation of DNA-PKcs at Ser2056 [21]; thus, p-DNA-PKcs foci were observed and used as an *in vivo* functional marker of NHEJ activity. Pretreating cells with either lactate isomer led to a significant increase in NCS-induced pSer2056-DNA-PKcs foci formation (Fig. 3). Stimulating cells with L- or D-lactate increased the percentage of p-DNA-PKcs-positive cells by 11 % and 7 %, respectively. Interestingly, lactate-driven enhancement of DNA-PKcs activation was also accompanied by higher DNA-PKcs nuclear immunoreactivity, indicating increased retention of protein in nucleus (Additional file 7). Taken together, these results demonstrate that lactate stimulates DNA-PKcs activity and suggests the substantial involvement of NHEJ in the lactate-induced enhancement of DNA repair.

### L- and D-lactate enhance cervical cancer cell survival after chemotherapeutic treatment

**Fig. 4:**
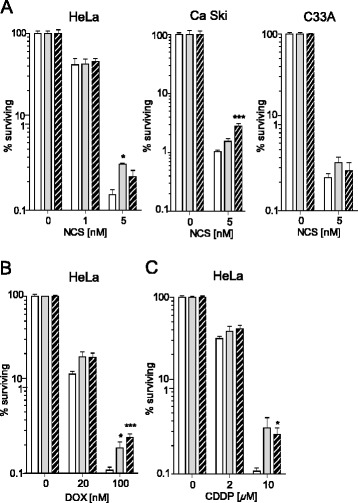
Lactate treatment enhances the resistance of cervical cancer cells to chemotherapeutics. Cells were incubated in the presence or absence of 20 mM L- or D-lactate for 24 h, followed by treatment with NCS (**a**), DOX (**b**) or CDDP (**c**) for the next 24 h in lactate-containing medium before seeding for colony formation in drug-free medium. The results are expressed as the means ± SEM of three independent experiments; cells treated with vehicle (*white columns*), cells treated with L-lactate (*grey columns*), cells treated with D-lactate (*striped columns*). Statistical significance was evaluated using one-way ANOVA followed by Tukey’s test. *P < 0.05, **P < 0.01 and ***P < 0.001 indicate significant differences compared to the untreated cells

Clonogenic survival assays were performed to investigate the effect of lactate on NCS-, DOX- and CDDP-mediated genotoxicity. HeLa cells were treated with 20 mM L- or D-lactate for 24 h, followed by incubation for 24 h in a DNA-damaging agent before seeding for colony formation. L- and D-lactate alone had no significant effect on HeLa cell survival (94 ± 7 % and 92 ± 10 % compared to the control, respectively). However, cell survival enhancement was observed when HeLa cells were exposed to chemotherapeutics in the presence of lactate (Fig. 4a, b and c). The protective effect of lactate on cell survival was most prominent at the highest NCS, DOX and CDDP concentrations tested in the assay (5 nM, 100 nM and 10 μM, respectively); these concentrations were used to calculate the survival increase factor (SIF) values summarised in Table 2. Surprisingly, the observed effects differed for specific chemotherapeutic and lactate isomer combinations. L-lactate exerted a greater protective effect against NCS, whereas D-lactate exerted a greater protective effect against DOX (Fig. 4 and Table 2). To confirm that the observed effect of lactate on the modulation of cellular resistance to chemotherapeutics is not restricted to HeLa cells, the clonogenic potential of Ca Ski and C33A cervical cancer cells was examined in the presence of lactate and NCS. As shown in Fig. 4a, both lactate isomers moderately enhanced the survival of these cervical cancer cell types. L-lactate treatment increased the survival of both Ca Ski and C33A cells treated with NCS by 1.5-fold, whereas D-lactate treatment increased the survival of Ca Ski and C33A cells by 1.2- and 2.7-fold, respectively.

**Table 2:** Modulation of the chemoresistance of cervical cancer cells by lactate

Cell line	Drug	SF^a^	SF_L_	SF_D_	SIF_L_	SIF_D_
HeLa	NCS	0.14 ± 0.02	0.33 ± 0.01^*^	0.23 ± 0.04	2.37 ± 0.44	1.66 ± 0.59
HeLa	DOX	0.10 ± 0.01	0.18 ± 0.04^*^	0.27 ± 0.00^***^	1.86 ± 0.52	2.77 ± 0.29
HeLa	CDDP	0.01 ± 0.001	0.033 ± 0.001	0.027 ± 0.006^*^	3.37 ± 1.42	2.79 ± 0.82
Ca Ski	NCS	1.04 ± 0.05	1.54 ± 0.18	2.82 ± 0.23^***^	1.49 ± 0.25	2.72 ± 0.36
C33A	NCS	0.23 ± 0.03	0.35 ± 0.06	0.28 ± 0.07	1.52 ± 0.44	1.21 ± 0.45

^a^ *SF* survival fraction for 5 nM NCS, 100 nM DOX or 10 μM CDDP; *SF*_*L,D*_ survival fraction after L-lactate or D-lactate pretreatment; *SIF* survival increase factor. The SIFs were calculated using the equation SIF_lactate_ = SF_lactate_/SF; the means of at least three independent experiments are reported. Statistical significance was evaluated using one-way ANOVA followed by Tukey’s test. *P < 0.05 and ***P < 0.001 indicate significant differences between SF_lactate_ and SF

### HCAR1 abundance and functionality in cervical cancer cell lines

**Fig. 5:**
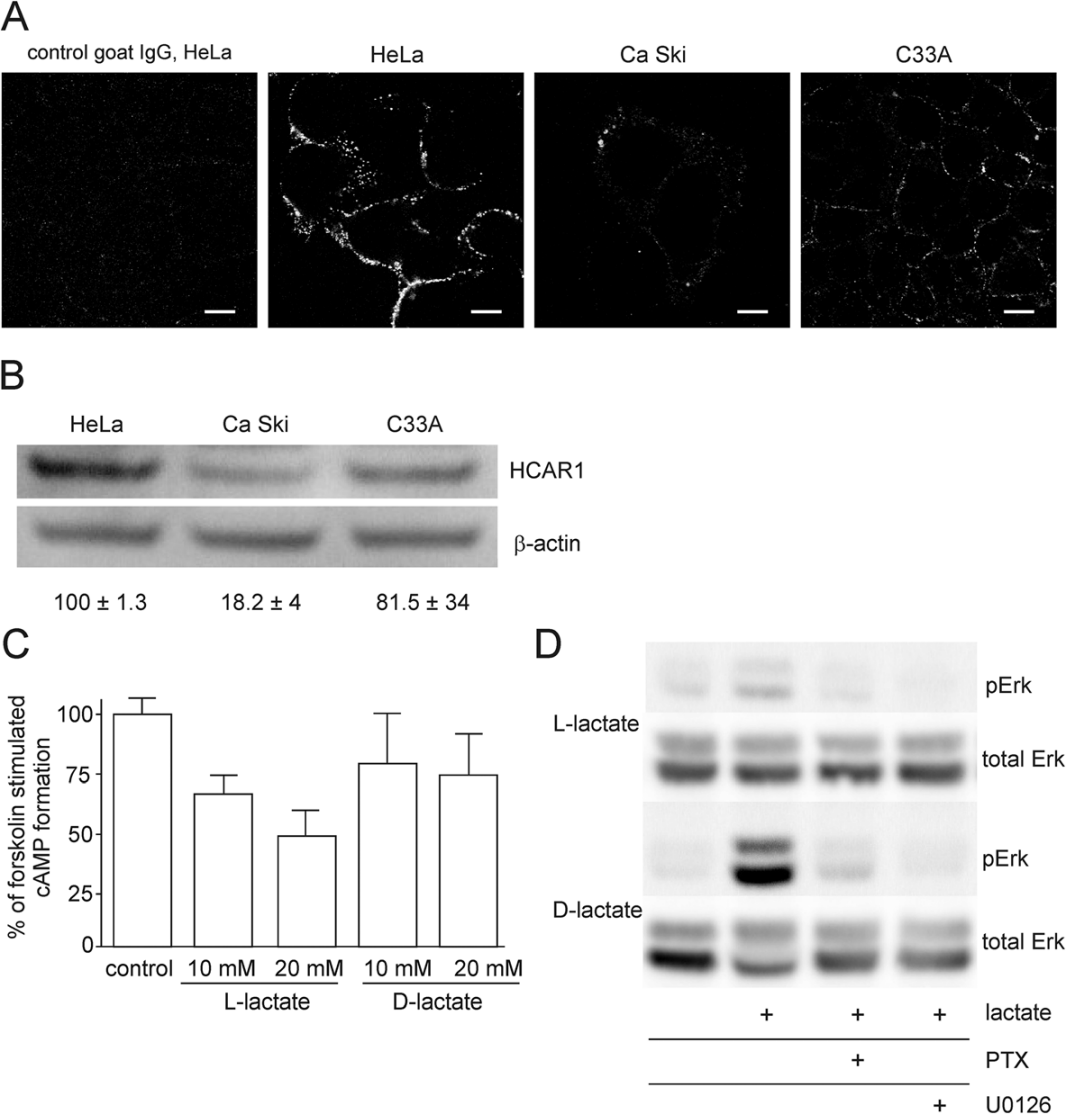
Cervical carcinoma cells express HCAR1 and respond to stimulation with lactate. **a** Immunocytochemical staining of HCAR1; scale bar, 10 μm. **b** Representative Western blot for the HCAR1 protein level in three cervical carcinoma cell lines (HeLa, Ca Ski and C33A). The HCAR1 protein level was quantified using densitometry and presented as relative means ± SEM of HCAR1/β-actin ratio normalised to HeLa cells, calculated from three independent experiments. **c** L- and D-lactate inhibit cAMP accumulation in forskolin-stimulated HeLa cells. Cyclic AMP was measured in cells treated with L- or D-lactate for 30 min in the presence of 10 μM forskolin. The results are expressed as the means ± SEM of at least three independent experiments. **d** Stimulation of Erk phosphorylation by L- or D-lactate is sensitive to PTX and U0126. Serum-starved HeLa cells were pretreated or not with PTX (100 ng/ml) or U0126 (10 μM) and stimulated with L- or D-lactate for 5 min. Representative blots of three independent experiments are shown

Recently, the presence of HCAR1 has been shown to be crucial for pancreatic and breast cancer cell survival [6, 7]. To evaluate the potential role of HCAR1 in the response of cervical cancer cells to chemotherapeutics, we first assessed the expression of this receptor in three cervical cell lines: HeLa, Ca Ski and C33A. The highest cellular HCAR1 immunoreactivity was found in HeLa cells (Fig. 5a), reflecting the HCAR1 protein level observed via Western blot (Fig. 5b). We confirmed the functionality of HCAR1 expressed in HeLa cells by assessing cAMP formation and Erk phosphorylation [22]. Because HCAR1 is a G_i/o_-coupled receptor that inhibits adenylate cyclase, we measured the inhibition of cAMP accumulation by lactate (10–20 mM) in forskolin-stimulated HeLa cells. We observed dose-dependent inhibition of cAMP formation by lactate treatment in forskolin-stimulated cells and found L-lactate to be a more active agonist than D-lactate (Fig. 5c). Stimulating the cells with L-lactate also triggered MAPK signalling (Fig. 5d) within 5 min, as described previously [22]. In contrast to its weak inhibition of cAMP accumulation, D-lactate strongly stimulated Erk phosphorylation compared to L-lactate, which showed moderate Erk pathway activation. These observations suggest that L- and D-lactate may differ in intrinsic activity towards their target receptors; this property is described in the literature as functional selectivity, or “bias”, towards certain response pathways [23]. G-protein coupled receptor-dependent MAPK pathway activation by lactate was confirmed by treatment of the cells with pertussis toxin (PTX), which uncouples G_i/o_ proteins from their receptors, and with a MAPK inhibitor. Pretreatment with either PTX or U0126 abolished lactate stimulated Erk phosphorylation (Fig. 5d).

### Pertussis toxin compromises L-lactate-, D-lactate- and 3,5-dihydroxybenzoic acid- stimulated γ-H2AX foci resolution in cells treated with chemotherapeutics

**Fig. 6:**
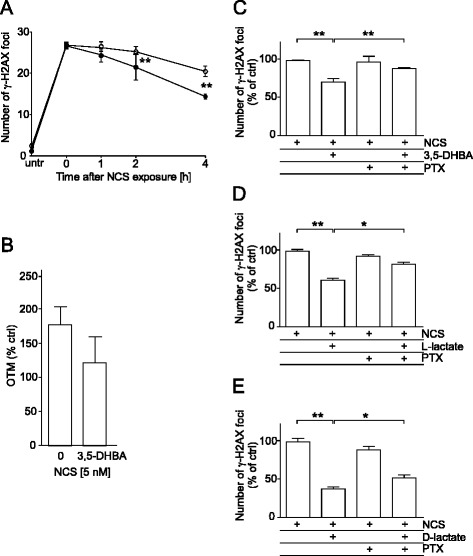
Stimulation of γ-H2AX foci resolution with L-lactate, D-lactate and 3,5-dihydroxybenzoic acid (3,5-DHBA) in cells treated with neocarzinostatin is compromised by pertussis toxin pretreatment. HeLa cells were incubated in the presence or absence of 500 μM 3,5-DHBA, 20 mM L-lactate or 20 mM D-lactate for 24 h, followed by treatment with 2 nM NCS for 30 min. Then, the cells were allowed to recover for 4 h unless otherwise indicated. **a** γ-H2AX foci resolution kinetics after 3,5-DHBA treatment. The graphs show the mean number of γ-H2AX foci per cell ± SEM from at least three independent experiments; drug alone (*white circles*), 3,5-DHBA + drug (*black circles*). **b** DSB repair after NCS treatment was measured by a neutral comet assay. HeLa cells were incubated in the presence or absence of 3,5-DHBA for 24 h, followed by treatment with 5 nM NCS for 30 min. Then, the cells were allowed to recover for 2 h. The basal Olive tail moment (OTM) was subtracted, and the value observed at 0 h was set to 100 %. The graph shows the mean OTM (% of control) ± SEM from three independent experiments. **c**, **d**, **e** γ-H2AX foci resolution in HeLa cells pretreated with 100 ng/ml PTX for 1 h before incubation in the presence or absence of 3,5-DHBA (**c**), L-lactate (**d**) or D-lactate (**e**). The graphs show the mean number of γ-H2AX foci per cell expressed as a % of control ± SEM from at least three independent experiments. Statistical significance was evaluated using Student’s *t*-test. *P < 0.05 and **P < 0.01 indicate significant differences compared to the untreated cells

To assess the role of HCAR1 in lactate-mediated stimulation of DNA repair, we studied the kinetics of γ-H2AX foci formation in HeLa cells incubated with the HCAR1-specific agonist 3,5-dihydroxybenzoic acid (3,5-DHBA) for 24 h followed by treatment with NCS. 3, 5-DHBA significantly increased the resolution of γ-H2AX foci (Fig. 6a) after treatment with NCS and its protective effect was confirmed by neutral comet assay results (Fig. 6b and Additional file 8). Furthermore, the inhibition of G_i/o_ proteins by PTX partially abolished the enhancement of γ-H2AX foci resolution due to 3, 5-DHBA treatment (Fig. 6c). Next, we evaluated the effects of PTX pretreatment on the disappearance of γ-H2AX foci in L- and D-lactate-treated cells exposed to NCS. Pretreatment with PTX significantly inhibited both the L- and D-lactate-induced disappearance of γ-H2AX foci in NCS-treated cells (Fig. 6d, e).

### HCAR1 and MCT activity is required for the L- and D-lactate-mediated enhancement of DNA repair and of HeLa cell survival

**Fig. 7:**
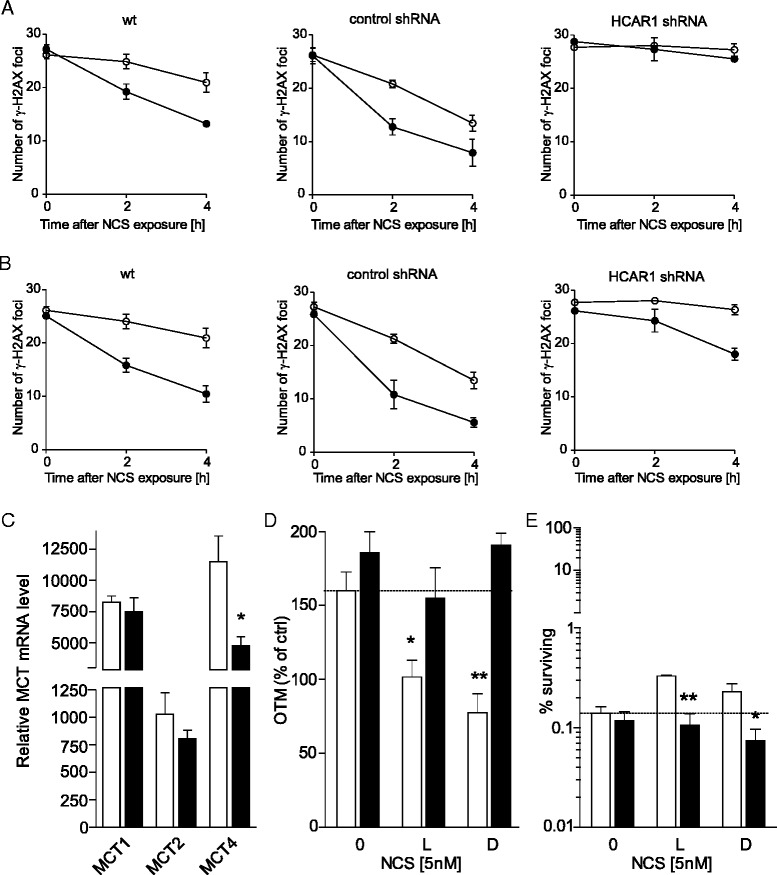
HCAR1 and MCT activity is required for the L- and D-lactate-mediated enhancement of DNA repair and cell survival. **a**, **b** γ-H2AX foci resolution kinetics in control and HCAR1 shRNA-expressing HeLa cells incubated in the presence or absence of (**a**) 20 mM L-lactate or (**b**) 20 mM D-lactate for 24 h and then treated with 2nM NCS for 30 min.; drug alone (*white circles*), lactate + drug (*black circles*). **c** The relative mRNA level of *MCT1*, *MCT2* and *MCT4* in control (*white column*) and HCAR1 shRNA-expressing HeLa cells (*black column*). **d**, **e** Pretreatment of HeLa cells with the MCT inhibitor α-CHCA abolishes the modulatory effect of lactate. **d** HeLa cells were pretreated with 2 mM α-CHCA for 1 h before incubation in the presence or absence of L-lactate (20 mM) or D-lactate (20 mM) for 24 h. Then, the cells were exposed to NCS (5 nM) for 30 min and allowed to recover for 2 h before harvesting for the neutral comet assay. The basal OTM was subtracted, and the value observed at 0 h was set to 100 %. The graph shows the mean OTM ± SEM from three independent experiments; cells treated with vehicle (*white columns*), cells treated with α-CHCA (*black columns*). **e** The clonogenic survival of HeLa cells pretreated with 2 mM α-CHCA for 1 h before incubation in the presence or absence of L-lactate (20 mM) or D-lactate (20 mM) for 24 h. Then, the cells were exposed to NCS (5 nM) for the next 24 h and seeded for colony formation in drug-free medium; cells treated with vehicle (*white columns*), cells treated with α-CHCA (*black columns*). Statistical significance was evaluated using Student’s *t*-test. *P < 0.05 and **P < 0.01 indicate significant differences compared to the corresponding counterparts. The results are expressed as the means ± SEM of three independent experiments

To further explore the mechanism by which HCAR1 is involved in the lactate-mediated enhancement of DNA repair and cervical carcinoma cell survival, we studied the kinetics of γ-H2AX foci formation in cells expressing reduced level of HCAR1 (Additional file 9). We performed series of experiments using wild type HeLa, control shRNA- or HCAR1 shRNA-expressing cells, incubated in the presence of either L- or D-lactate and treated with NCS. The resultant data showed that silencing *HCAR1* expression exerted profound effects on DNA repair kinetics in these cells (Fig. 7a, b). We observed a considerable decrease in DNA repair efficiency in the HCAR1 shRNA-expressing cells compared to the control shRNA-expressing cells in the presence or absence of lactate. HCAR1 knockdown abolished the L-lactate-induced enhancement of DNA repair (Fig. 7a) and decreased the stimulatory effects of D-lactate on DNA repair (Fig. 7b). The present evidence indicates a correlation of HCAR1 and MCT genes expression with cancer cell survival in the tumour microenvironment [6]. Subsequently, we evaluated the functional relevance of MCTs to the lactate-mediated enhancement of DNA repair. We found that cells expressing shRNA against HCAR1 expressed 10, 21 and 60 % lower mRNA levels of MCT1, MCT2 and MCT4, respectively, compared to control shRNA-expressing cells (Fig. 7c). Finally, we performed clonogenic and comet assays in the presence of α-cyano-4-hydroxycinnamic acid (α-CHCA), a classic MCT inhibitor [3], to confirm whether the observed stimulatory effects of lactate on DNA repair and cell survival depend on intracellular lactate. HeLa cells were treated with α-CHCA (2 mM) for 1 h before exposure to NCS with or without lactate. α-CHCA pretreatment had no significant effect on basal DNA repair or cell survival after exposure to NCS. However, α-CHCA abolished the L- and D-lactate-mediated increases in DNA repair after 5 nM NCS exposure as evaluated by the comet assay (Fig. 7d). Furthermore, α-CHCA significantly suppressed the protective effects of L- and D-lactate on cell survival after NCS treatment (Fig. 7e). Our results suggest that extracellular lactate transport by MCTs coordinated by HCAR1 is responsible for the observed effects of lactate on the enhancement of DNA repair and cell survival.

## Discussion

Recent evidence has identified lactate as an active metabolite and a pseudo-hormone that coordinates metabolic processes at the systemic and cellular levels. L-lactate produced by malignant tumours may be a crucial component enabling significant cancer cell growth and resistance [6, 7, 24]. Latham and colleagues have demonstrated that both L- and D-lactate inhibit HDACs and promote changes in gene expression in a manner similar to the established HDAC inhibitors butyrate and trichostatin A [9]. This evidence strongly suggests that lactate is involved in the posttranslational modifications of histone and non-histone proteins and that lactate participates in the modulation of protein signalling and activity. Our study is the first to demonstrate that pretreating cervical cancer cells with lactate improves their DNA repair capacity and enhances cell survival following chemotherapeutic treatment. In the present study, we incubated cervical cancer cells with 10–20 mM lactate to mimic physiological lactate concentrations in the lower female genital tract environment. Lower female genital tract epithelium-associated bacterial flora are the major producers of the L- and D-lactate found in vaginal secretions (at concentrations as high as 10–50 mM). Biochemical experiments demonstrated that incubating HeLa cells with L- or D-lactate decreased the activity of class I and II HDACs in living cells as expected. The inhibition of HDACs by lactate was accompanied by the acetylation of histones H3 and H4 and by a decrease in DNA compactness (Fig. 1). Incubating cells with lactate for 24 h evoked a transcriptionally permissive chromatin conformational state and a subsequent significant up-regulation of important genes involved in DNA DSB repair, including *LIG4*, *NBS1*, and *APTX* (Table 1). Indeed, mutation of the genes encoding DNA ligase IV (*LIG4*), Nibrin (*NBS1*) or Aprataxin (*APTX*) results in DDR disorders, specifically LIG4 syndrome, Nijmegen breakage syndrome (NBS) and Ataxia oculomotor apraxia-1, in association with increased radiosensitivity [25–27]. Recent evidence suggests that HATs and HDACs are recruited to DNA DSB sites to create a repair-proficient chromatin state that orchestrates the activity of repair and signalling proteins, thereby promoting DNA repair processes [12–14]. Our study is the first to demonstrate that L- and D-lactate stimulate the activation of DNA-PKcs (Fig. 3), the essential component of NHEJ, although previous studies have demonstrated that histone H3 acetylation induced by trichostatin A [20] or histone acetylation in response to DSBs [15] facilitates DNA-PKcs activation. Thus, it is conceivable that such up-regulation of DNA repair mechanisms via a lactate-elicited increase in the activity of DNA-PKcs and in the transcriptional expression of DNA ligase IV, Nibrin and Aprataxin could translate into accelerated processing of DNA damage. Indeed, L- and D-lactate improved the kinetics of γ-H2AX foci formation and resolution and the dynamics of DNA DSB repair after exposure to NCS, DOX or CDDP. Both lactate isomers significantly enhanced DNA repair, although D-lactate, a stronger HDAC inhibitor, was found more effective than L-lactate (Fig. 2). The observations of lactate-induced enhancement of DNA repair were further supported by the protective effects of lactate on cell survival. We showed that lactate increased the survival of three cervical cancer cell lines: HeLa, Ca Ski and C33A; however, the Ca Ski and C33A cell lines were less prone to lactate-induced modulation than the HeLa cell line (Fig. 4). Interestingly, the enhancement of cervical cancer cell survival by L-lactate treatment corresponded to the HCAR1 protein level in the respective cell lines (Fig. 5a, b). Of the three examined cell lines, the HeLa cell line, demonstrating the most abundant expression of HCAR1, showed the most prominent protective effect of lactate on clonogenic survival, as its survival fraction (SF) increased by 2.4-fold after L-lactate treatment, which was higher than that of the Ca Ski and C33A cell lines (both 1.5-fold) (Table 2). Our observations are in line with recent evidence indicating that the HCAR1 levels correlate to the rates of cancer tumour growth and metastasis [6]. In the present study, we demonstrated that cervical cancer cell lines display HCAR1 surface expression and that both lactate isomers induced signalling pathways in a receptor-dependent fashion (Fig. 5a-d). Interestingly, L-lactate preferentially stimulated Gi-mediated pathway, inhibited forskolin-induced intracellular cAMP and slightly activated MAPK pathway, while D-lactate triggered MAPK pathway only. These observation suggest that L- and D-lactate may differ in intrinsic activity towards HCAR1 resulting in differential activation of signal transduction pathways associated with this receptor [23]. In addition, incubating HeLa cells with the HCAR1 agonist 3,5-DHBA improved the kinetics of γ-H2AX foci formation and resolution to a similar but smaller extent than lactate. As expected, uncoupling G-proteins from HCAR1 via pretreatment with PTX decreased L-lactate-, D-lactate- and 3,5-DHBA-stimulated γ-H2AX foci disappearance in cells exposed to NCS (Fig. 6c-e). Furthermore, experiments using HCAR1 shRNA-expressing cells showed that silencing HCAR1 exerted profound effects on DNA repair kinetics, which were observed as considerably diminished γ-H2AX foci disappearance kinetics in the presence or absence of lactate (Fig. 7a, b). Based on the study by Roland and co-workers [6] HCAR1 is implicated in the regulation of lactate transport mechanisms. Our study using HCAR1 shRNA-expressing HeLa cells revealed that silencing HCAR1 affects the mRNA level of MCT4 (Fig. 7c), which facilitate the cellular uptake of lactate. Complimentary experiments performed using α-CHCA, a classic MCT inhibitor [3], confirmed that the observed stimulatory effect of lactate on DNA repair and cell survival depends on its intracellular activity (Fig. 7d, e). Taken together, these data demonstrated that lactate transport by MCTs is crucial for its intracellular activity, which leads to chromatin rearrangement and enhanced DNA repair and cell survival. However, although the observed lactate-induced stimulation of DNA repair activity seems to result from its inhibitory activity on HDACs, we cannot rule out the possibility that other HCAR1-dependent responses may affect cellular DNA repair capacity. It is worth noted that D-lactate induced marked Erk phosphorylation via PTX-sensitive pathway compared to slight MAPK signalling activation by L-lactate (Fig. 5d). A detailed study by Li and co-workers [22] showed that upon HCAR1 activation, the dissociation of the G_βγ_ subunit from the G_i_ protein subsequently induces Erk activation via two distinct pathways: a PKC-dependent pathway and an IGF-1R transactivation-dependent pathway [22]. Because IGF-1R and EGFR are known for their cross-talk [28] and both receptors are involved in HR and NHEJ [28, 29], the involvement of the HCAR1/IGF-1R/EGFR axis in DNA repair requires further investigation.

## Conclusions

In the present study, we reported a new potential mechanism underlying the interaction between lower female genital tract microbiota and cervical epithelial cells under physiological conditions. Vaginal and ectocervical microbiota not only protect against pathogen colonisation by acidifying the mucosa using lactic acid but also, according to our results, appear to modulate the activity of the cervical cancer cells in a manner that alters its resistance to chemotherapeutics. Our data indicate a novel mechanism by which lactate modulates the cellular DNA damage repair process in cervical cancer cells; in this mechanism, L- and D-lactate are actively transported across the cell membrane by MCTs to intracellular compartments, leading to the inhibition of class I and II HDACs. This inhibition results in the hyperacetylation of histones H3 and H4, chromatin relaxation, DNA repair gene up-regulation and DNA-PKcs activation in the nucleus. Thus, lactate creates a DNA repair-proficient environment that stimulates DNA repair dynamics and significantly enhances cervical carcinoma cell survival after drug treatment. Furthermore, we also showed that lactate-induced DNA repair enhancement is regulated by the HCAR1/MCT axis, as lactate receptor down-regulation or MCT inhibition notably affects DNA repair efficiency. We suggest that the enhancement of DNA repair machinery activity by lactate may account for the increased resistance of malignant cervical tumours to standard clinical therapy (such as cisplatin and ionising radiation). Thus, targeting lactate-mediated signalling in cervical cancer environment, e.g. by locally delivered MCTs inhibitors and/or HCAR1 antagonist, might improve efficacy of anticancer therapy.

## Materials and methods

### Chemicals

All chemicals were purchased from Sigma-Aldrich (St. Louis, MO, USA) unless otherwise stated. Doxorubicin, forskolin, α-CHCA and U0126 were dissolved in anhydrous DMSO and added to cells at a final DMSO concentration of 0.5 % (*v/v*). Control cells were incubated in 0.5 % DMSO alone.

### Cell culture

The HeLa, Ca Ski and C33A human cervical cancer cell lines were purchased from American Type Culture Collection (ATCC, Manassas, VA, USA) and were authenticated by short tandem repeat profiling (LGC Standards, UK) at the end of the study. HeLa cells were cultured in DMEM, and Ca Ski and C33A cells were cultured in RPMI medium (Life Technologies, Carlsbad, CA, USA) supplemented with 10 % foetal bovine serum (PAA Laboratories GmbH, Pasching, Austria) and antibiotics (Life Technologies) at 37 °C in a humidified atmosphere containing 5 % CO_2_. The cells were routinely tested for mycoplasma contamination and were passaged every 3 days using TrypLE Express (Life Technologies).

### HDAC assay

The inhibitory effects of lactate and butyrate on cellular HDAC activity were measured using an HDAC-Glo I/II Assay Kit (Promega, Madison, WI, USA) for class I/II HDACs according to the manufacturer’s protocol. Briefly, HeLa cells grown on 96-well plates were treated with sodium L-lactate, sodium D-lactate, sodium butyrate or sodium chloride for 2 h. Then, HDAC-Glo™ I/II Reagent was added, and the luminescence was directly measured using a chemiluminescence plate reader after a 30-min incubation at room temperature.

### Western blot analysis

The histone acetylation status of HeLa cells was evaluated after 24 h incubation in lactate or butyrate. The harvested cells were washed twice with ice-cold phosphate-buffered saline (PBS) supplemented with either lactate (10 or 20 mM) or butyrate (5 mM) and centrifuged at 400 *g* for 8 min at 4 °C. Then, the cells were suspended in Triton extraction buffer (TEB: PBS containing 0.5 % Triton X-100 (*v/v*), 2 mM PMSF, and 0.02 % (*v/v*) NaN_3_) at a density of 1 × 10^6^ cells/100 μl and lysed on ice for 10 min. After centrifugation at 6500 *g* for 10 min, the pellet was washed with a half volume of TEB, suspended in 0.2 N HCl at a density of 3 × 10^6^ cells/70 μl, and incubated at 4 °C overnight for acidic extraction. The next day, the samples were centrifuged, and the extracts were neutralised with a 1/50 volume of 10 N NaOH, followed by SDS-PAGE (NuPAGE gradient gel 4–12 %, Life Technologies) and transfer to a nitrocellulose membrane. After 1 h of blocking (SuperBlock Blocking Buffer, Thermo Fisher Scientific, Inc., Waltham, MA, USA) and overnight incubation at 4 °C in anti-acetyl-H3 and -H4 primary antibodies (#39140 and #39967, Active Motif, Carlsbad, CA, USA), the membrane was washed with TBS and incubated in an HRP-conjugated secondary antibody (Dako, Ely, UK) for 1 h at RT. Antibody binding was visualised via chemiluminescence using SuperSignal West Pico Substrate (Thermo Fisher Scientific, Inc.). The membrane was stripped and re-probed with anti-H3 and -H4 primary antibodies (#39763 and #61300, Active Motif) for 1 h at RT as described above. The experimental design for evaluating the HCAR1 protein level and Erk phosphorylation was as described above with minor changes. For analysis of the HCAR1 protein level, cells grown at 70 % confluence were harvested using a cell scraper, centrifuged and lysed with RIPA buffer supplemented with a protease inhibitor cocktail (Roche Diagnostics GmbH). For Western blot analysis of Erk phosphorylation, HeLa cells that were serum-starved (DMEM, 0.5 % FBS) overnight were stimulated with lactate for 5 min in the presence or absence of 100 ng/ml PTX (overnight pretreatment) or 10 μM of the MAPK inhibitor U0126 (2 h pretreatment). The cells were harvested using a cell scraper, centrifuged and lysed with PhosphoSafe Extraction Reagent (Novagen, EMD Chemicals, CA, USA) containing a protease inhibitor cocktail (Roche). The primary antibodies used were anti-GPR81 (ab106942, Abcam, Cambridge, UK), anti-p-Erk1/2, anti-Erk and anti-β-actin conjugated to HRP (sc-101760, sc-94 and sc-1616, respectively, Santa Cruz Biotechnology, Inc.). The membrane handling and incubation conditions were as described above. Chemiluminescence signals were captured and quantified using a G:BOX gel imager (Syngene, Cambridge, UK).

### Quantification of DNA compactness

Cells grown on a 96-well plate were washed with ice-cold PBS, fixed in 4 % formaldehyde for 20 min and washed twice with PBS. After the final PBS wash, the cells were stained with 1 μg/ml of Hoechst 33,342 solution in PBS for 10 min and subjected to analysis using an ArrayScan VTI HCS Reader (Thermo Fisher Scientific, Inc.) equipped with a 40× objective. To visually assess changes in chromatin structure after lactate or butyrate treatment, representative images were subjected to Sobel edge detection and thresholding using ImageJ software. The process of chromatin expansion decreases the number of distinct spaces within the nucleus, and these changes can be visualised via the detection of the Sobel edges as shown by Irianto and co-workers [30]. Quantitative analysis of chromatin compactness was performed by calculating the CV for the Hoechst intensity of all pixels within individual nuclei according to the method described by Contrepois and co-workers [31]. The Target Activation Bioapplication software (Thermo Fisher Scientific, Inc.) was set up to analyse 100 cells per well and to calculate the mean fluorescence and standard deviation of Hoechst staining within each nucleus. The CV parameter was obtained by dividing the standard deviation by the mean fluorescence. Each experiment was performed in six replicates.

### DNA DSB repair assay

DNA DSB repair was measured via a neutral comet assay as previously described [32] using a Comet Assay Kit (Trevigen, Gaithersburg, MD, USA). Comets were stained with SYBR Green I, visualised using a Nikon D-Eclipse Ti fluorescence microscope (5× objective) and analysed using CASP software [33].

### Colony formation assay

Actively growing cells in flasks were incubated in the presence or absence of lactate for 24 h before treatment with chemotherapeutics for the next 24 h. Then, the cells were harvested via trypsinisation, seeded in 10-cm-diameter Petri dishes at densities ranging from 500 to 400,000 per dish in triplicate in drug-free medium and then allowed to form colonies. After 14 days, the colonies were fixed in Carnoy’s solution and stained with crystal violet. Images of the dishes were captured using a G:BOX imager and processed using ImageJ software, which was set up to count colonies containing > 50 cells. The SIF was calculated by dividing the SF of cells treated with a cytotoxic agent and lactate by the SF of cells treated with a cytotoxic agent alone. The SF values used to determine the SIF were calculated using the linear quadratic model [SF = exp(−aD-bD2)] in GraphPad Prism software according to the least-squares fit.

### γ-H2AX, phospho-DNA-PKcs and HCAR1 immunocytochemistry

Cells grown on a 96-well plate were washed with ice-cold PBS and fixed with ice-cold methanol:acetone (1:1) for 20 min at −20 °C. After the blocking procedure (1 % BSA in PBST, 1 h), the cells were stained with a rabbit antibody against γ-H2AX (ab2893, Abcam, Cambridge, UK) at 4 °C overnight in a humidified chamber. Primary antibody binding was visualised using an Alexa Fluor 594-conjugated goat anti-rabbit antibody (Life Technologies) followed by nuclear staining with 1 μg/ml Hoechst 33342 for 20 min. The plate was analysed using an ArrayScan VTI HCS Reader equipped with a 40× objective. Images of 20 fields per well were routinely acquired, and 150 cells/well were analysed using Spot Detector Bioapplication V3 software. Each experiment was performed in triplicate. The experimental design for evaluating DNA-PKcs phosphorylation was as described above with minor changes. The cells were fixed (4 % formaldehyde, 20 min), permeabilised (0.25 % Triton X-100 in PBS, 10 min) and blocked (3 % BSA in PBST, 30 min) before incubation in an anti-pS2056-DNA-PKcs antibody (ab18192, Abcam) as the primary antibody overnight at 4 °C. Images were acquired using an ArrayScan VTI HCS Reader equipped with a 20× objective and analysed using Spot Detector Bioapplication V3 software (p-DNA-PKcs, 250 cells/well). Each experiment was performed in six replicates. For cell surface HCAR1 staining, cells grown on Lab-Tek chamber slides (Nunc, Thermo Fisher Scientific, Inc.) were washed with HBSS and incubated in an anti-FKSG80 antibody (sc-32647, Santa Cruz Biotechnology, Inc.) in HBSS at 4 °C for 60 min. After fixation with 4 % formaldehyde and blocking with 5 % normal donkey serum in PBS for 30 min, the cells were incubated in an Alexa Fluor 488-conjugated anti-goat secondary antibody (Invitrogen) at RT for 1 h and stained with Hoechst 33342. Images were acquired using a confocal microscope (Nikon D-Eclipse C-1 Plus) equipped with a 63× objective.

### Real time-PCR

Total RNA was extracted from cells using TRIzol reagent (Sigma-Aldrich) according to the manufacturer’s instructions. Then, total RNA (5 μg) was reverse-transcribed using a Maxima First Strand cDNA Synthesis Kit for RT-qPCR (Thermo Fisher Scientific, Inc.). PCR was performed using LightCycler 480 SYBR Green I Master Mix (Roche Diagnostics GmbH, Mannheim, Germany) and a Roche LightCycler 480 Instrument (Roche Diagnostics GmbH). Relative gene expression was normalised to the housekeeping genes hydroxymethylbilane synthase (*HMBS*) and hypoxanthine phosphoribosyl transferase (*HPRT*) and were calculated using the ΔΔCt method. In total, 5 reference genes (*GAPDH*, *ACTB*, *HPRT*, *HBMS*, *TBP*) were tested and Normfinder [34] was used to identify the most stably expressed housekeeping genes (stability values: *GAPDH*: 0.153, *ACTB*: 0.240, *HPRT*: 0.091, *HBMS*: 0.095, *TBP*: 0.115).The study of mRNA expression included the following genes: *LIG3*, *XRCC1*, *PNKP*, *PARP1*, *PARP2*, *RAD51*, *BRCA1*, *BRCA2*, *RAD50*, *MRE11A*, *NBS1*, *XRCC6*, *XRCC5*, *PRKDC*, *LIG4*, *XRCC4*, *DCLRE1C*, *WRN*, *NHEJ1*, *ATM*, *ATR*, *TP53*, *APTX*, *PARD3*, *MDC1, MCT1, MCT2, MCT4,* and *HCAR1*. The primer sequences are listed in Additional file 10.

### cAMP accumulation assay

The day before the experiment, the culture medium was replaced with serum-free medium. All experiments were conducted in the presence of the phosphodiesterase inhibitor IBMX at 500 μM. For the cAMP accumulation studies, HeLa cells were treated with 10–20 mM L- or D-lactate in the presence of forskolin (10 μM) to stimulate cAMP synthesis. The reaction was incubated at 37 °C for 30 min and then terminated via two cold PBS washes, immediately followed by cell harvesting using a cell scraper. The centrifuged cells were resuspended in lysis buffer, and the measurement of the intracellular cAMP levels was performed using a cAMP assay kit (R&D Systems). The data are presented as the means ± SEM of at least three separate experiments.

### Short hairpin RNA

shRNA (Dharmacon, Lafayette, CO, USA) was used to silence HCAR1 according to the protocol provided by the manufacturer. Briefly, 24 h before transfection, HeLa cells were seeded on 24-well plates at a density of 5 × 10^4^ cells/well. Next, the cells were transfected with 1 μg of HCAR1 shRNA plasmid DNA using 2 μl of TurboFect Transfection Reagent (Life Technologies) in serum-free DMEM. Control cells were treated with non-targeted shRNA (Dharmacon). The next day, the medium was replaced with DMEM containing 10 % FBS and puromycin (7.5 μg/ml) (Life Technologies), and the selection of cells expressing the transgene was continued for 3 weeks, and the selection medium was changed every 3 days. Resultant puromycin-resistant and GFP-positive cells were evaluated for *HCAR1* expression via real-time PCR and Western blot.

### Statistical analysis

The experiments were repeated at least three times, each of which was conducted in three or six replicates (except for Western blot analysis). The data are presented as the means ± SEM. GraphPad Prism software was used to analyse and plot the data. Statistical significance was evaluated using Student’s *t*-test or one-way ANOVA followed by Tukey’s test.

## Additional files

Additional file 1: Figure S1A. γ-H2AX foci immunolabelling after neocarzinostatin treatment. Cells were incubated in the presence or absence of 20 mM L- or D-lactate for 24 h, followed by treatment with 2 nM for 30 min. Then, the cells were allowed to recover for the indicated period prior to fixation and γ-H2AX staining. Representative images for the respective treatments are shown. (PDF 255 kb) Additional file 2: Figure S1B. γ-H2AX foci immunolabelling after doxorubicin treatment. Cells were incubated in the presence or absence of 20 mM L- or D-lactate for 24 h, followed by treatment with 2 μM DOX for 30 min. Then, the cells were allowed to recover for the indicated period prior to fixation and γ-H2AX staining. Representative images for the respective treatments are shown. (PDF 456 kb) Additional file 3: Figure S1C. γ-H2AX foci immunolabelling after cisplatin treatment. Cells were incubated in the presence or absence of 20 mM L- or D-lactate for 24 h, followed by treatment with 20 μM CDDP for 30 min. Then, the cells were allowed to recover for the indicated period prior to fixation and γ-H2AX staining. Representative images for the respective treatments are shown. (PDF 322 kb) Additional file 4: Figure S2A. DNA repair in HeLa cells after exposure to neocarzinostatin. Cells were incubated in the presence or absence of 20 mM L- or D-lactate for 24 h, followed by treatment with 5 nM NCS for 30 min. Then, the cells were allowed to recover for the indicated period prior to harvesting for the neutral comet assay. Each image shows a representative area of a microscopic slide for the particular treatment from the same experiment. (PDF 156 kb) Additional file 5: Figure S2B. DNA repair in HeLa cells after exposure to doxorubicin. Cells were incubated in the presence or absence of 20 mM L- or D-lactate for 24 h, followed by treatment with 2 μM DOX for 30 min. Then, the cells were allowed to recover for the indicated period prior to harvesting for the neutral comet assay. Each image shows a representative area of a microscopic slide for the particular treatment from the same experiment. (PDF 123 kb) Additional file 6: Figure S2C. DNA repair in HeLa cells after exposure to cisplatin. Cells were incubated in the presence or absence of 20 mM L- or D-lactate for 24 h, followed by treatment with 20 μM CDDP for 30 min. Then, the cells were allowed to recover for the indicated period prior to harvesting for the neutral comet assay. Each image shows a representative area of a microscopic slide for the particular treatment from the same experiment. (PDF 140 kb) Additional file 7: Figure S3. Hela cells incubated in the presence of L-lactate or D-lactate show higher nuclear DNA-PKcs immunoreactivity. HeLa cells were incubated in the presence or absence of 20 mM L-lactate or 20 mM D-lactate for 24 h. Then, fixed and permeabilised cells were stained with anti-DNA-PKcs antibody (sc-9051, Santa Cruz Biotechnology, Inc.), followed by incubation with Alexa Fluor 594-conjugated secondary antibodies. The data are averaged from three independent experiments and presented as the means ± SEM of the percentage of cells exhibiting greater DNA-PKcs nuclear immunoreactivity than the untreated cell population in three independent experiments. **P* < 0.05 and ***P* < 0.01 indicate significant differences compared to the untreated cells. (PDF 206 kb) Additional file 8: Figure S4. DNA repair in HeLa cells incubated with 3,5-DHBA. Cells were incubated in the presence or absence of 500 μM 3,5-DHBA for 24 h and followed by treatment with NCS (5 nM) for 30 min. Then, the cells were allowed to recover for 2 h prior to harvesting for the neutral comet assay. Each image shows a representative area of a microscopic slide for the particular treatment from the same experiment. (PDF 83 kb) Additional file 9: Figure S5. Efficacy of the shRNA against HCAR1. (A) Quantification of the mRNA expression of HCAR1 in wild type, control shRNA- and HCAR1 shRNA-expressing HeLa cells. (B) Western blot for HCAR1 protein expression in wild type, control shRNA- and HCAR1 shRNA-expressing HeLa cells. (PDF 68 kb) Additional file 10: Table S1. Sequences of the primers used for real-time PCR. (PDF 8 kb)
