# Supplementary figures and images for: L- and D-lactate enhance DNA repair and modulate the resistance of cervical carcinoma cells to anticancer drugs via histone deacetylase inhibition and hydroxycarboxylic acid receptor 1 activation
Source: Cell Commun Signal. 2015 Jul 25;13:36. doi: 10.1186/s12964-015-0114-x (PMC4514991; doi:10.1186/s12964-015-0114-x)

Figure S1A

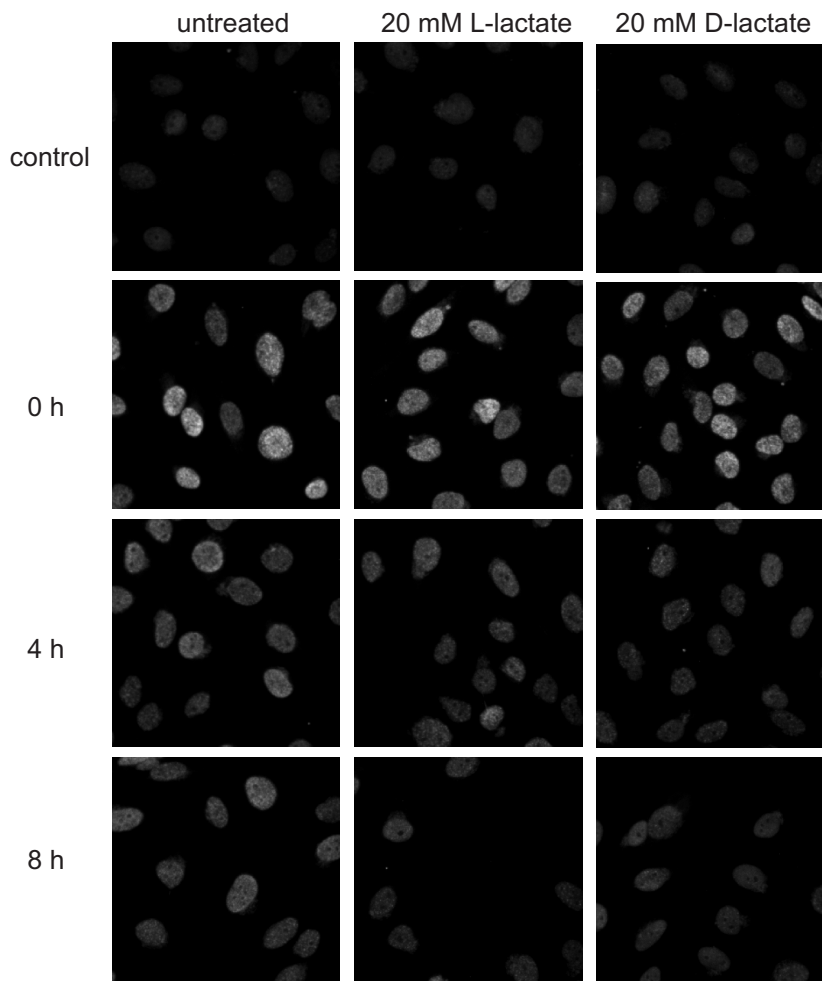

Supplement: Additional file 1: Figure S1A. — γ-H2AX foci immunolabelling after neocarzinostatin treatment. Cells were incubated in the presence or absence of 20 mM L- or D-lactate for 24 h, followed by treatment with 2 nM for 30 min. Then, the cells were allowed to recover for the indicated period prior to fixation and γ-H2AX staining. Representative images for the respective treatments are shown. (PDF 255 kb) [file 12964_2015_114_MOESM1_ESM.pdf]

Figure S1B

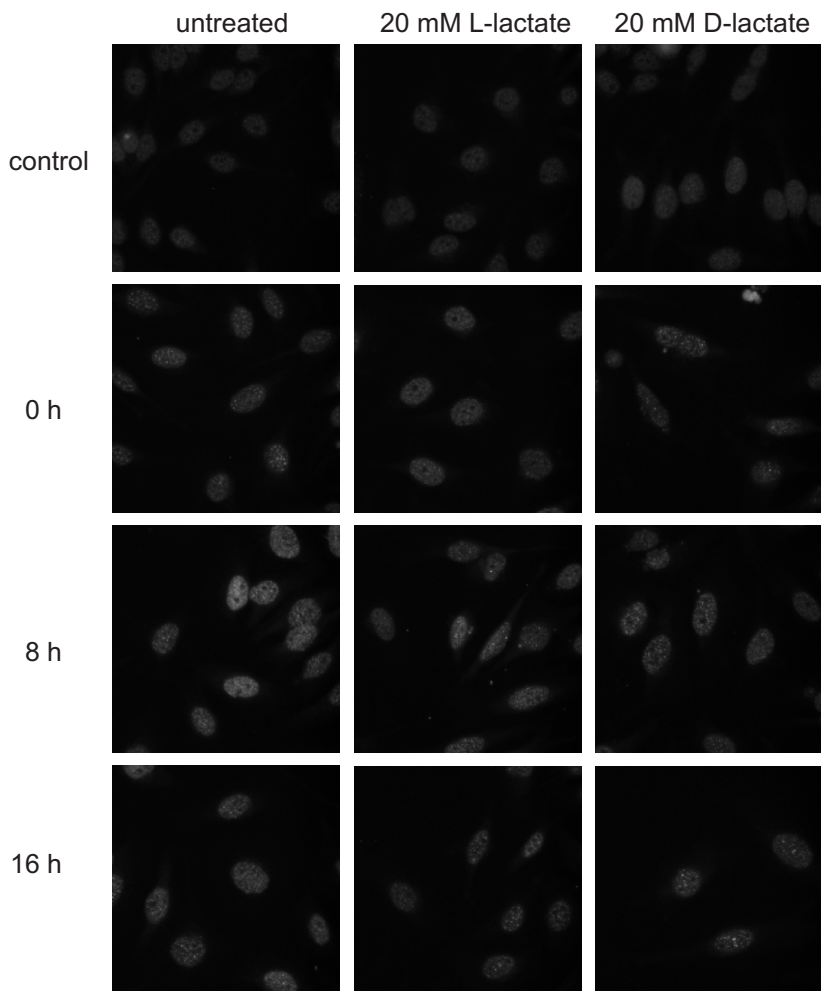

Supplement: Additional file 2: Figure S1B. — γ-H2AX foci immunolabelling after doxorubicin treatment. Cells were incubated in the presence or absence of 20 mM L- or D-lactate for 24 h, followed by treatment with 2 μM DOX for 30 min. Then, the cells were allowed to recover for the indicated period prior to fixation and γ-H2AX staining. Representative images for the respective treatments are shown. (PDF 456 kb) [file 12964_2015_114_MOESM2_ESM.pdf]

Figure S1C

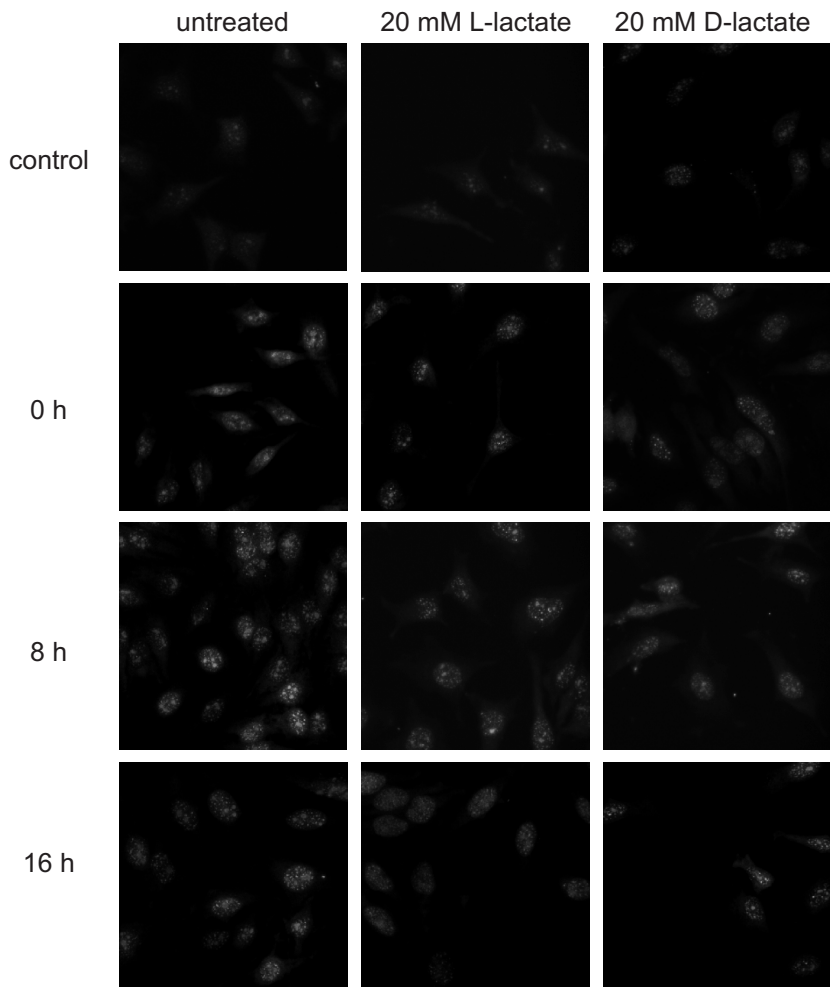

Supplement: Additional file 3: Figure S1C. — γ-H2AX foci immunolabelling after cisplatin treatment. Cells were incubated in the presence or absence of 20 mM L- or D-lactate for 24 h, followed by treatment with 20 μM CDDP for 30 min. Then, the cells were allowed to recover for the indicated period prior to fixation and γ-H2AX staining. Representative images for the respective treatments are shown. (PDF 322 kb) [file 12964_2015_114_MOESM3_ESM.pdf]

Figure S2A

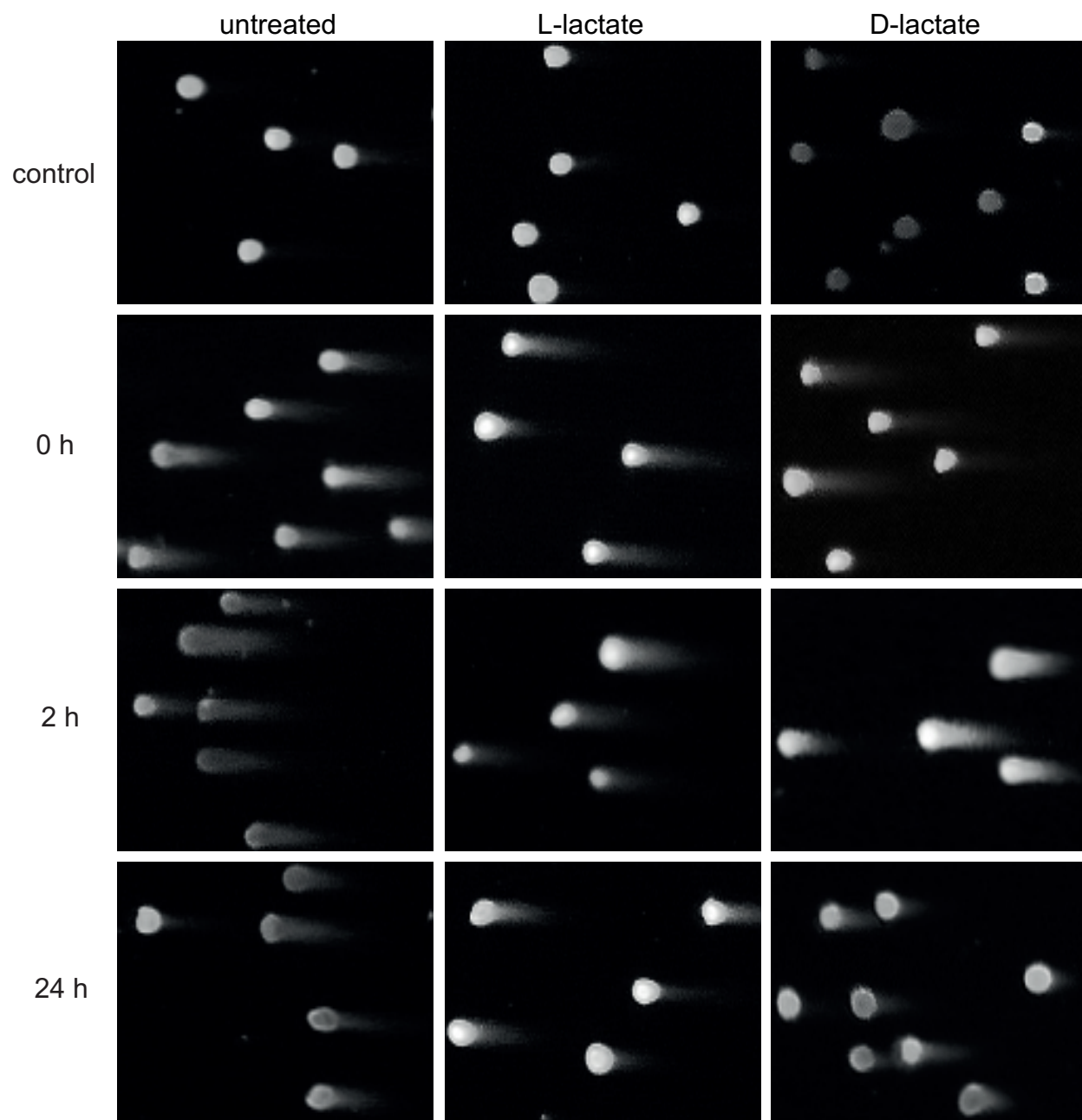

Supplement: Additional file 4: Figure S2A. — DNA repair in HeLa cells after exposure to neocarzinostatin. Cells were incubated in the presence or absence of 20 mM L- or D-lactate for 24 h, followed by treatment with 5 nM NCS for 30 min. Then, the cells were allowed to recover for the indicated period prior to harvesting for the neutral comet assay. Each image shows a representative area of a microscopic slide for the particular treatment from the same experiment. (PDF 156 kb) [file 12964_2015_114_MOESM4_ESM.pdf]

Figure S2B

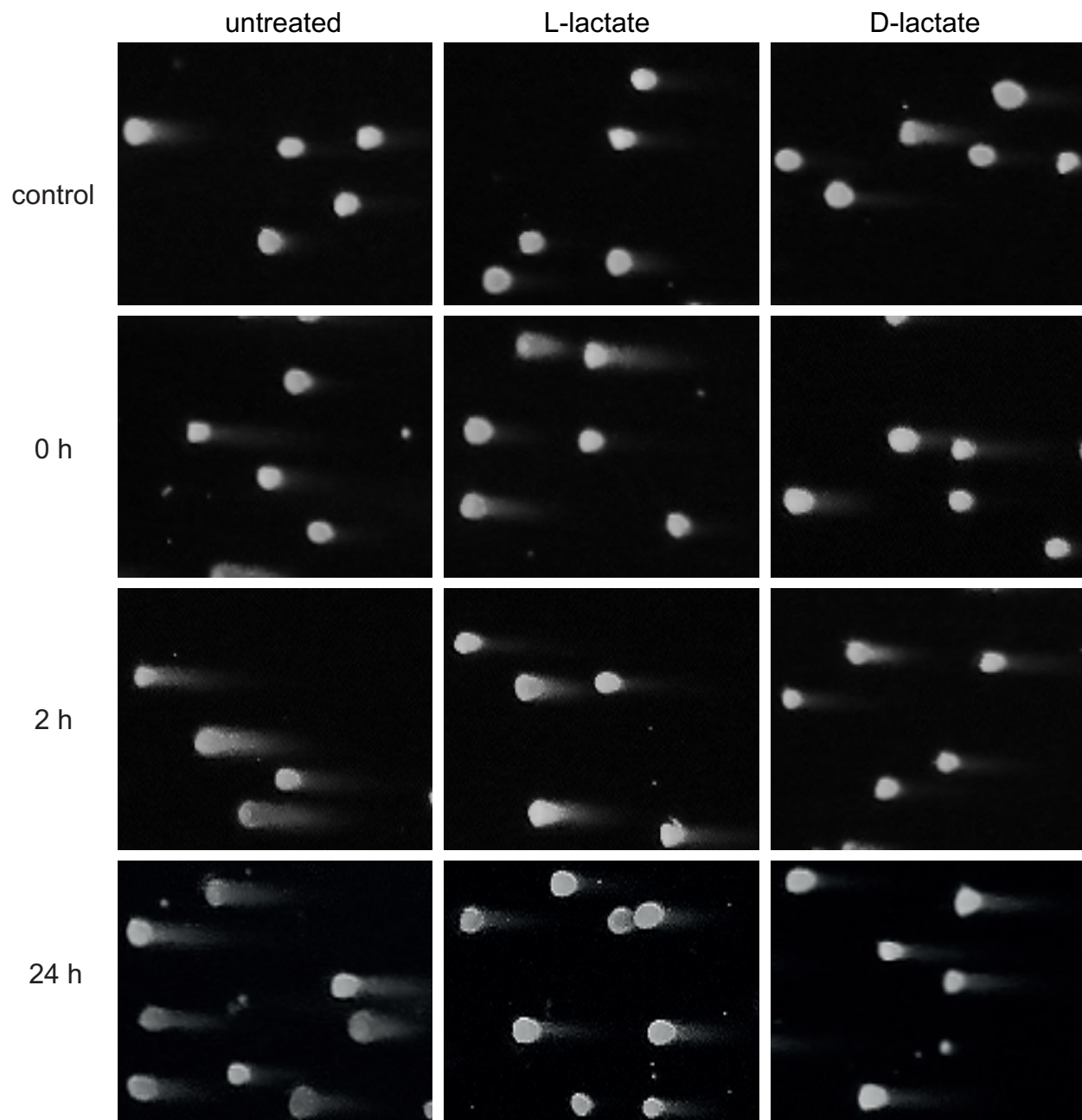

Supplement: Additional file 5: Figure S2B. — DNA repair in HeLa cells after exposure to doxorubicin. Cells were incubated in the presence or absence of 20 mM L- or D-lactate for 24 h, followed by treatment with 2 μM DOX for 30 min. Then, the cells were allowed to recover for the indicated period prior to harvesting for the neutral comet assay. Each image shows a representative area of a microscopic slide for the particular treatment from the same experiment. (PDF 123 kb) [file 12964_2015_114_MOESM5_ESM.pdf]

Figure S2C

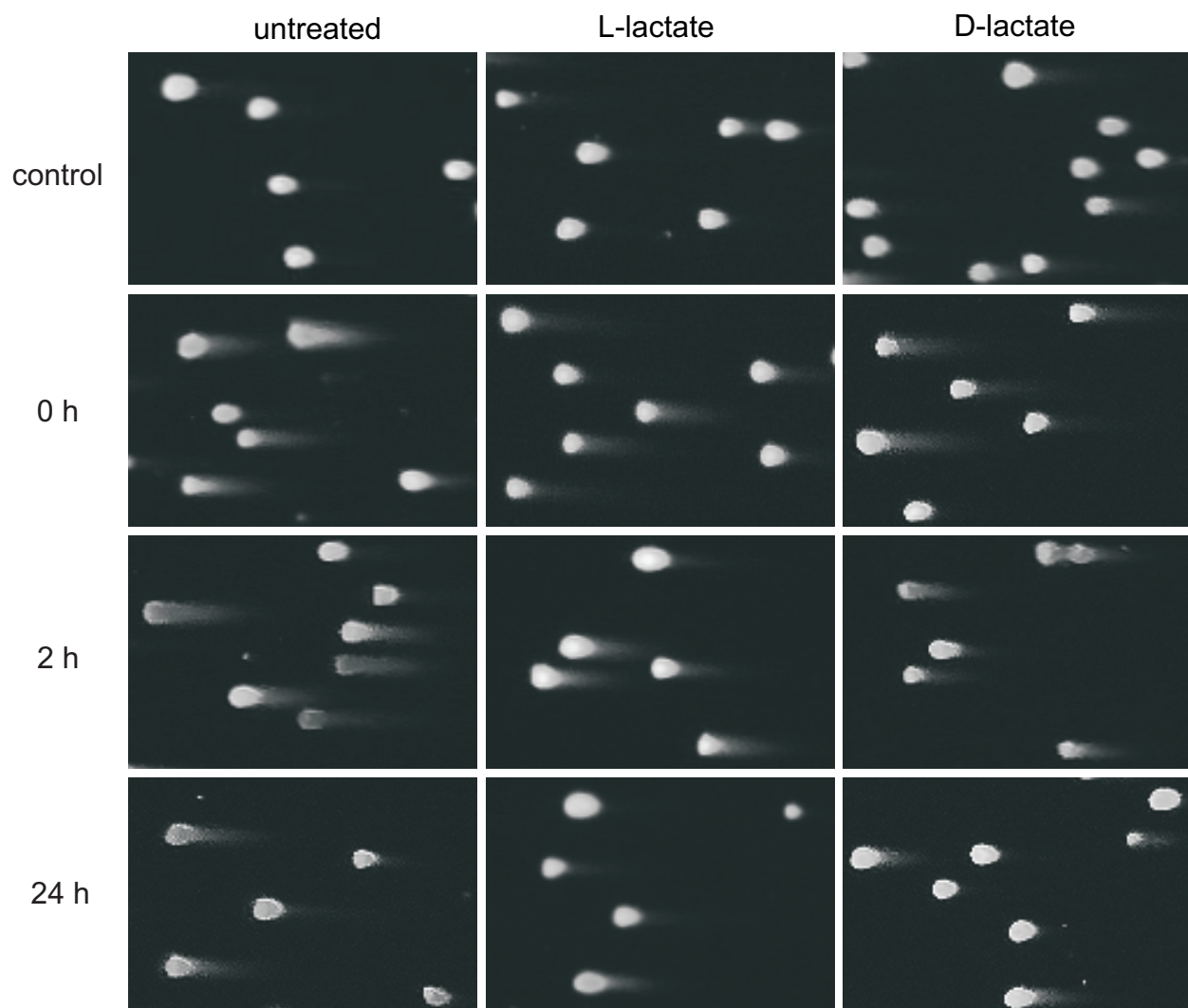

Supplement: Additional file 6: Figure S2C. — DNA repair in HeLa cells after exposure to cisplatin. Cells were incubated in the presence or absence of 20 mM L- or D-lactate for 24 h, followed by treatment with 20 μM CDDP for 30 min. Then, the cells were allowed to recover for the indicated period prior to harvesting for the neutral comet assay. Each image shows a representative area of a microscopic slide for the particular treatment from the same experiment. (PDF 140 kb) [file 12964_2015_114_MOESM6_ESM.pdf]

# Figure S3

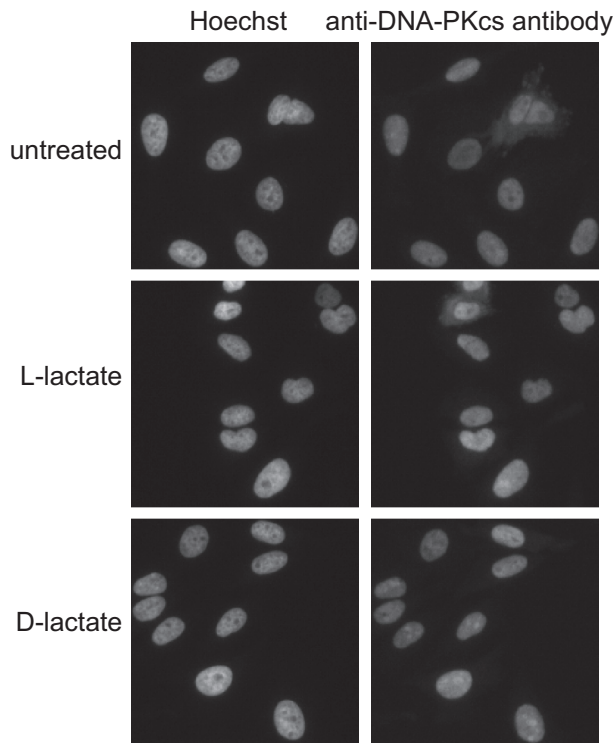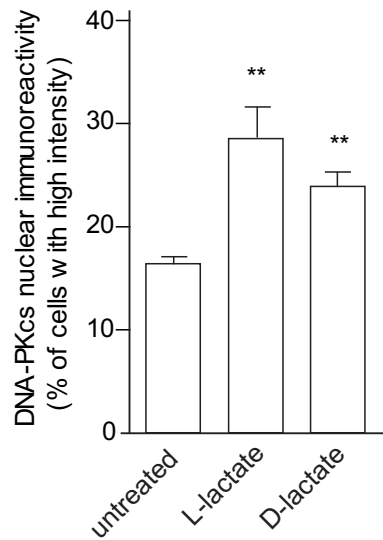

Supplement: Additional file 7: Figure S3. — Hela cells incubated in the presence of L-lactate or D-lactate show higher nuclear DNA-PKcs immunoreactivity. HeLa cells were incubated in the presence or absence of 20 mM L-lactate or 20 mM D-lactate for 24 h. Then, fixed and permeabilised cells were stained with anti-DNA-PKcs antibody (sc-9051, Santa Cruz Biotechnology, Inc.), followed by incubation with Alexa Fluor 594-conjugated secondary antibodies. The data are averaged from three independent experiments and presented as the means ± SEM of the percentage of cells exhibiting greater DNA-PKcs nuclear immunoreactivity than the untreated cell population in three independent experiments. *P < 0.05 and **P < 0.01 indicate significant differences compared to the untreated cells. (PDF 206 kb) [file 12964_2015_114_MOESM7_ESM.pdf]

Figure S4

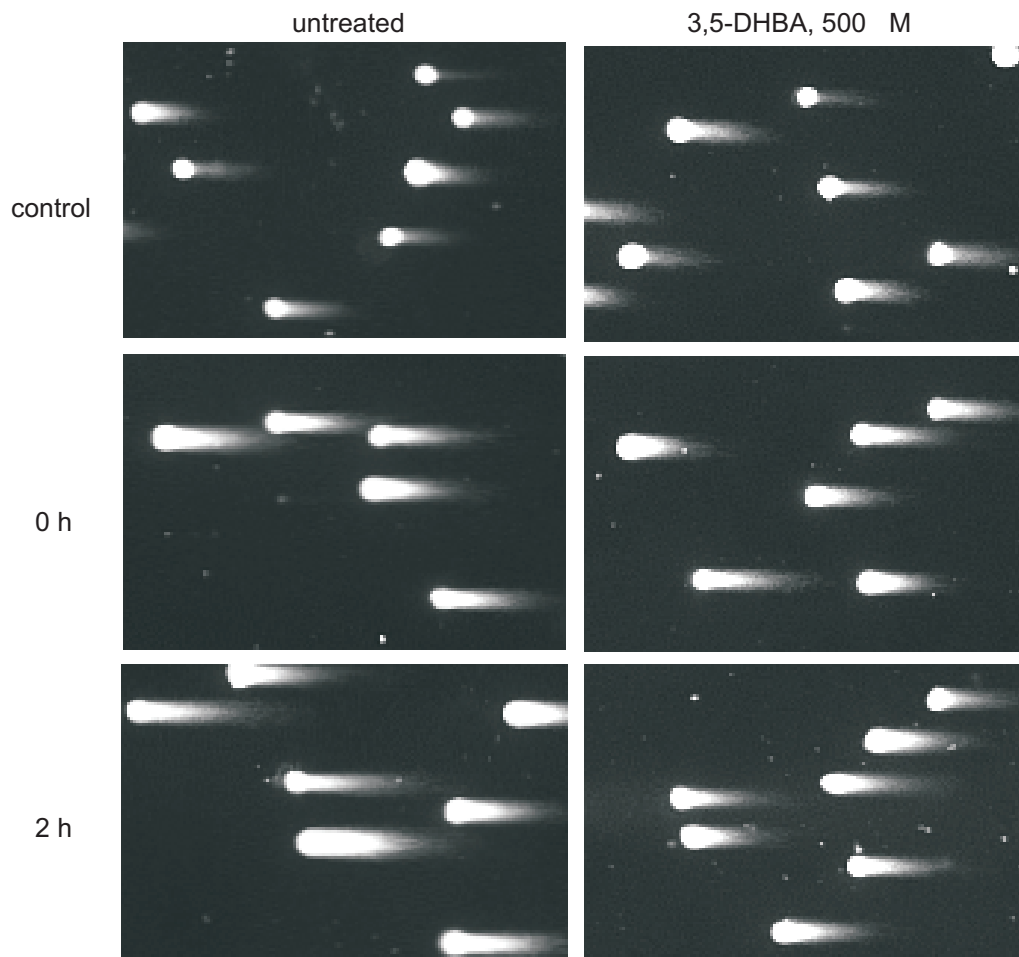

Supplement: Additional file 8: Figure S4. — DNA repair in HeLa cells incubated with 3,5-DHBA. Cells were incubated in the presence or absence of 500 μM 3,5-DHBA for 24 h and followed by treatment with NCS (5 nM) for 30 min. Then, the cells were allowed to recover for 2 h prior to harvesting for the neutral comet assay. Each image shows a representative area of a microscopic slide for the particular treatment from the same experiment. (PDF 83 kb) [file 12964_2015_114_MOESM8_ESM.pdf]

# Figure S5

## A

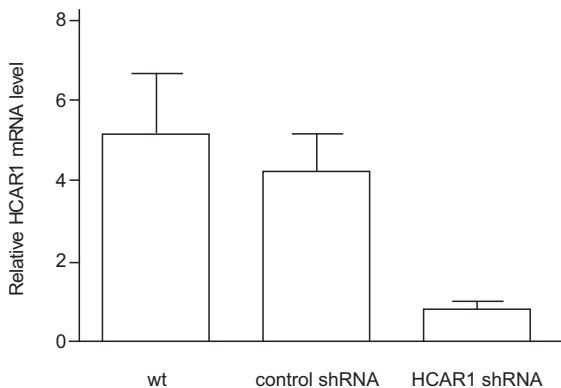

## B

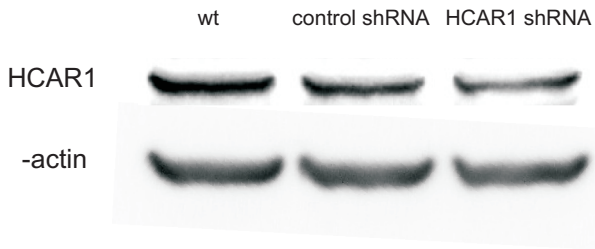

Supplement: Additional file 9: Figure S5. — Efficacy of the shRNA against HCAR1. (A) Quantification of the mRNA expression of HCAR1 in wild type, control shRNA- and HCAR1 shRNA-expressing HeLa cells. (B) Western blot for HCAR1 protein expression in wild type, control shRNA- and HCAR1 shRNA-expressing HeLa cells. (PDF 68 kb) [file 12964_2015_114_MOESM9_ESM.pdf]
