# Supplementary material for: L- and D-lactate enhance DNA repair and modulate the resistance of cervical carcinoma cells to anticancer drugs via histone deacetylase inhibition and hydroxycarboxylic acid receptor 1 activation
Source: Cell Commun Signal. 2015 Jul 25;13:36. doi: 10.1186/s12964-015-0114-x (PMC4514991; doi:10.1186/s12964-015-0114-x)
Supplement: Additional file 10: Table S1. — Sequences of the primers used for real-time PCR. (PDF 8 kb) [file 12964_2015_114_MOESM10_ESM.pdf]

Table S1. Sequences of the primers used in real-time PCR.

**Gene**	**Forward Primer 5'-3'**	**Reverse Primer 5'-3'**
LIG3	AAGGGAAGCTGAGTAACTCCA	CCGCCTCTTTGTTTGGCAC
XRCC1	CTTTGGCTTGAGTTTTGTACGG	TGGGGATGTCTTGTTGATCCG
PNKP	ACAACAACCGGTTTCGAGAG	ATCTCCAGGATGGCAGAGAA
PARP1	GATGCCTATTACTGCACTGGG	CGGTCCTGCTTTTTAACCTTCAA
PARP2	TGATGACGTCAGCGTTCG	TTCTTGGCAGGGGAAGAGT
RAD51	ATCACTAATCAGGTGGTAGCTCAA	CCCCTCTTCCTTTCCTCAGA
BRCA1	CTTAGAGTGTCCCATCTGTCTGG	GCCCTTTCTTCTGGTTGAGA
BRCA2	GCGCGGTTTTTGTCAGCTTA	TGGTCCTAAATCTGCTTTGTTGC
RAD50	GCTTCTGATAAAAGGCGGAAT	GCGAATGATGAGTGAGGCTAA
MRE11A	AAGATGATGAAGTCCGTGAGG	TCATTAGCCATCTGTTCTGCTAAA
NBS1	CACTCACCTTGTCATGGTATCAG	CTGCTTCTTGGACTCAACTGC
XRCC6	AGTCATATTACAAAACCGAG	CCTTGGAGGCATCAACCAAA
XRCC5	CAACAAAAAGTCAGATTCCTCTCTC	GCTGTAGGTCCATCTTCATGGT
PRKDC	CCAAGAGATCTTCCTTCTTGGA	CGTAAGGGCGAAAGACCTC
LIG4	GCTTCTCCTACTGAAGGTTCTCT	TGCAGATCAGAATCCTCTACCA
XRCC4	AATCCACCTTGTTTCTGAACCC	CCTTTTTCCATTGCCATGTCATC
DCLRE1C	ACTCGGAGCCCGTACCAT	TGTCTAGCTTATTCACATGA
WRN	CCAATTTTATTTCTCCGAGGATCT	GATCAGCTGACGGGAAAAAG
NHEJ1	CTTCAACAGGTGTGGCATGA	ACAATGGGCGAAGGAGATTA
ATM	GCAGCTGGAAGAAGCACAA	TTTTAGGCTGGGATTGTTCG
ATR	TGTAGAGAGATGGAGACCAACG	GACCAATCGGTTGACTTCTGA
TP53	TTCTGTCCCTTCCCAGAAAA	GTTGGCAAAACATCTTGTTGAG
APTX	GCTACCACGCCATTCCGAG	CGAAGGGGCAGCTTCAAGAG
PARD3	GGAACTGTGAGCCTTCTGGT	AATATCCTCATCTTCTGCTTTCGT
MDC1	TGTGGGAGCCTTAATGGTACT	CAGAGCAAGTCAGCAAAGAGAA
MCT1	GTGGCTCAGCTCCGTATTGT	GAGCCGACCTAAAAGTGGTG
MCT2	CAACACCATTCCAAGACAGC	TGGCTGTTATGTACGCAGGA
MCT4	CAGTTCGAGGTGCTCATGG	ATGTAGACGTGGGTCGCAT
HCAR1	TGCCCAGCGTGTCTGCTAGACT	TACACCAGGGGATCCAGCATGC
